# Does Socioeconomic Deprivation Lead to More Drug-related Deaths?

**DOI:** 10.1192/bjo.2025.10130

**Published:** 2025-06-20

**Authors:** Fred Halliday, Caroline Copeland, Soraya Mayet

**Affiliations:** ^1^Hull York Medical School, York, United Kingdom; ^2^Kings College London, London, United Kingdom; ^3^Humber Teaching NHS FT, Hull, United Kingdom

## Abstract

**Aims:** Drug-related deaths are a tragedy, with socioeconomic deprivation associated with higher rates. Globally deaths have increased with most involving an opioid. We aimed to assess the rates and causes of drug-related deaths for a deprived city in Northern England, compared with the surrounding less deprived semirural county (with pockets of high deprivation) against national data. We want to assess whether there is an association of deaths with higher deprivation levels.

**Methods:** Drug-related deaths in 2022 were provided by Dr Copeland via the National Programme of Substance Use Mortality (NPSUM) using postmortem (PM) records. Two deaths did not have full postcodes, so not included where location was required. We assessed deaths against demographics, implicated drugs, prescribed medications, comparing with Indices of Multiple Deprivation (IMD) by postcode. Regional deaths were compared with Office for National Statistics (ONS) death rates. Statistical analysis via Excel.

**Results:** In 2022, there were 91 deaths for the city and county (14.8/100,000) significantly higher than (8.14/100,000) in England and Wales (X²=16.4, p<0.00001). The city had significantly more deaths (N=67;25.0/100,000, X²=95.6, p<0.00001) versus the county (N=18;5.2/100,000). Mean age of death 43.2±9.0 and 23% were women. Most deaths in the county occurred in urban areas. Median age of death for Males was 42.6 yrs. and Females 45.2 yrs. (SD±9.0). Most implicated drug causing death was heroin and morphine (23.1%), methadone (16.5%) like national data, whilst benzodiazepines (15.4%) were higher than national (p>0.05). Most deaths were caused by more than one implicated drug 82.4%. 64.8% of deaths occurred in a person known to be using drugs. Many deaths had methadone implicated (N=28; 30.8%) and 50 deaths had methadone at PM of which 11 were prescribed. Most deaths (N=55; 64.7%) occurred in the top decile of IMD and occurred in the top 4.4% most deprived neighbourhoods.

**Conclusion:** Socioeconomic deprivation was associated with higher rates of drug-related deaths; most deaths occurred in the most deprived areas. Addressing deprivation-related risks in economically challenged areas is critical to effectively tackling drug deaths and health inequalities. The cause of death was most often opioids and strategies such as take-home naloxone and optimising opioid substitution treatment will be vital to reduce deaths. We note a high proportion of deaths had methadone, which was not prescribed, present at postmortem, indicating that prescribed methadone may have been diverted. Drug services may consider strategies to increase use of supervised consumption as per guidelines to reduce diversion and associated deaths.

